# Reducing Carbon Footprint of Disposable Pulse Lavage Systems in Total Hip and Knee Arthroplasty

**DOI:** 10.7759/cureus.52195

**Published:** 2024-01-13

**Authors:** Matthew Chan, Jonathan Mutimer

**Affiliations:** 1 Trauma & Orthopaedics, Gloucestershire Hospitals National Health Service (NHS) Foundation Trust, Gloucester, GBR

**Keywords:** green surgery, total knee arthroplasty (tka), total hip athroplasty, pulse lavage, healthcare sustainability, carbon footprint

## Abstract

Pulse lavage is recommended in all modern total joint arthroplasty operations in the UK. The common current pulse lavage is a disposable battery-operated system. AC and power tool powered models are commercially available in the UK. We performed a carbon emissions analysis of each model to evaluate the reduction in the carbon footprint of the arthroplasty operations at one trust and extrapolated the data to scale the possible economic and environmental benefits. Introducing a power tool driven pulse lavage system can reduce the carbon footprint of pulse lavage by 50% compared to the battery and AC-powered options. Additionally, we have reduced the economic impact of one trust by switching to a “greener” alternative pulse lavage system. In trusts where a power tool-driven pulse lavage is not possible, we advocate using AC-powered kits that are less wasteful than the more commonly used battery-powered options.

## Introduction

Pulse lavage is used widely in orthopaedic surgery. It plays an important role in modern cementing techniques for total joint arthroplasty by ensuring a clean cancellous bone bed to allow cement inter-digitation [1-3]. For this reason, it has become part of the “gold standard” of modern cementing techniques. Additionally, it also plays a role in providing a high-volume washout of soft tissues during wound irrigation [4]. A variety of different disposable pulse lavage systems are available commercially. These differ mostly in the source of power (battery/alternating current (AC)), plastic/carbon footprint, and cost. Data from the National Joint Registry (NJR) showed our district general hospital performed over 1800 arthroplasty cases in 2019 [5], whilst nationally, it is estimated that 215,000 - 440,000 total hip and knee replacements will be performed by 2035 [6]. Consequently, thousands of disposable pulse lavage systems are used and discarded per year. It is therefore important to consider the significant environmental and economic implications for pulse lavage systems that are commonly used in these operations.

We aim to reduce the carbon footprint of the arthroplasty operations performed at our trust by reviewing the environmental implications of the common commercially available disposable pulse lavage systems. Additionally, we will consider the clinical, and economic impact of these devices.

## Materials and methods

Pulse lavage systems

Three main types of disposable pulse lavage systems are available on the market. These predominantly differ in their source of power. The first option commonly used is battery-powered (Pulsvac Plus by Zimmer-Biomet, Warsaw, US). Eight AA batteries are used to power this system, which then requires disposal at the end of the operation. The second option is an AC-powered version of the Pulsvac Plus. This model has an adaptor that allows it to be attached to the main supply in the operation theatre. The obvious advantage of this model is that it does not require the disposal of batteries. The last option is Ecopulse (De Soutter Medical Ltd., Aylesbury, UK). This novel system is powered using the power tool handpiece available on most arthroplasty sets. The motor from the power tool provides the propulsion of fluid so no motors are required in the product itself. Consequently, raw materials are required compared to the battery and AC-powered options.

Environmental analysis

Calculating the carbon footprint of each system requires details on the raw materials, their weights, and how the product is manufactured, distributed, and disposed. These details were sought from the manufacturer. Emissions factors are provided by the UK Government's GHG conversion factor report [7] and are used to calculate the carbon footprint of each product. An emissions factor is a ratio of the amount of carbon emissions (CO2e) generated and the outputs of the production processes. Each emission factor is unique to the specific raw material or process involved. The carbon footprint for the production of each material could be estimated by multiplying the weight of the materials used by their corresponding emissions factor.

$$\large Carbon\, footprint\, for\, production = Emission\, factor\, of\, raw\, material (KgCO2e)\, x\, Weight (Kg)$$

The carbon emissions for transportation were estimated using the distance travelled by each product from the distribution centre to our trust and multiplying this by the emissions factor for the vehicle used (e.g., heavy goods vehicle (HGV)).

$\large Carbon\, footprint\, for\, transportation = Emissions\, factor \, for \, vehicle \, (KgCO2e)\, x \, Distance \, travelled (Km)$

Lastly, the carbon emissions for disposal of the instrument were calculated by multiplying the weight by the emissions factor for disposal of that material.

$\large Carbon\, footprint\, for \, disposal = Emissions\, factor \, for \, disposal (KgCO2e)\, x\, Weight (Kg)$

The details of the factors used in this calculation are highlighted in Table 1. Adding the total carbon emissions of each of these factors provided a total estimated carbon footprint for each product.

**Table 1:** Factors used in carbon footprint analysis The emissions factor is a ratio of the amount of carbon emissions (CO2e) generated and the outputs of the production processes.

Factors used in carbon footprint analysis	Pulse lavage system			
Main components of the instrument	Emissions Factor (KgCO_^2^_e/T)	Ecopulse®	Pulsvac® (Battery)	Pulsvac® (AC)
Hard plastic main body	3413.08	Yes	Yes	Yes
Batteries	4633.48	No	Yes	No
Tubing	3760.00	Yes	Yes	Yes
Inner Packaging	4032.39	Yes	Yes	Yes
Outer packaging	919.4	Yes	Yes	Yes
Transport				
Distance from distribution centre to Hospital	Ferry = 0.016142 ; HGV = 0.22916	Yes	Yes	Yes

Economic analysis

The economic analysis was estimated by our procurement department, and an annual savings report was created. Due to the Non-Disclosure Agreement, exact figures were not available. However, list prices were provided by manufacturers and compared.

Clinical analysis

Finally, a product evaluation survey of the power tool driven model was completed by our surgical team. The trial period ran over two weeks, and the power tool driven pulse lavage system was used in both hip and knee arthroplasty.

## Results

Environmental analysis

The total annual carbon emissions are summarised in Table 2. These figures are based on the 1800 arthroplasty cases we performed in 2019. The power tool operated system produced around half as much carbon emissions compared to the battery and AC-powered counterparts. The main reason for this arises due to the differences in the power source. The lack of motors in the power tool drive system meant it was much lighter resulting in a lower carbon emission calculation. Additionally, having a simpler design likely leads to a more efficient manufacturing process. However, we have been unable to quantify this. Another important factor was in the transportation. The power tool operated system is manufactured and distributed in the UK whilst the battery/AC options are distributed from the manufacturers global distribution centre in the Netherlands. The added distance led to around a 7x increase in carbon emissions from transportation alone. This highlights the importance of considering where the product is made and distributed when evaluating new medical devices.

**Table 2:** Comparison of carbon emissions from disposable pulse lavage system

	Carbon Emissions (Tonnes CO_2_e)
Ecopulse (De Soutter, Aylesbury, UK)	3.0
Pulsvac Plus Battery (Zimmer-Biomet, Warsaw, US)	7.8
Pulsvac Plus AC (Zimmer-Biomet, Warsaw, US)	5.2

It is important to note that the AC-powered system had around 1/3 less carbon emissions as the battery-operated system. Again, this is likely due to the lack of batteries and the reduction in weight of this model. It highlights that battery-powered options are the least environmentally friendly of all pulse lavage models.

Economic analysis

**Table 3:** Comparison of Retail Prices

	Retail Price
Ecopulse (De Soutter, Aylesbury, UK)	£38
Pulsvac Plus Battery (Zimmer-Biomet, Warsaw, US)	£131
Pulsvac Plus AC (Zimmer-Biomet, Warsaw, US)	£191

A comparison of retail prices is highlighted in Table 3. This highlights that the power tool driven pulse lavage kit retails significantly less than the other models available on the market. The manufacturer suggests this is due to a reduction in raw materials and the simplicity of the design. However, these prices are list pricing on single units, and they will likely significantly change when trusts procurement team discuss the cost with the manufacturers. Factors such as volume and pre-existing manufacturer commitments may provide better pricing at your trust. We recommend a thorough discussion with all the manufacturers to get a more accurate cost analysis. Our procurement department has estimated the annual cost savings based on the purchase of 2,500 pulse lavage kits per year at our trust. We project that the power tool operated system would save £6,500 per year compared to the battery-powered option.

Clinical

The power tool operated system favoured well in the clinical trial period and was acceptable for most surgeons. Some of the advantages that were highlighted were that it was much quieter than the other options and hence made communication and training easier. One of the disadvantages is that once attached to the power tool, the overall weight was greater than the battery/AC-powered counterparts, making handling more difficult. In addition, with the power tool in use, mechanical brushes could not be used simultaneously to clear the femoral canal of debris. Consequently, it is likely our surgeons will continue to use battery/AC-powered pulse lavage for longer and more difficult operations such as revision surgery.

Other factors

**Figure 1:**
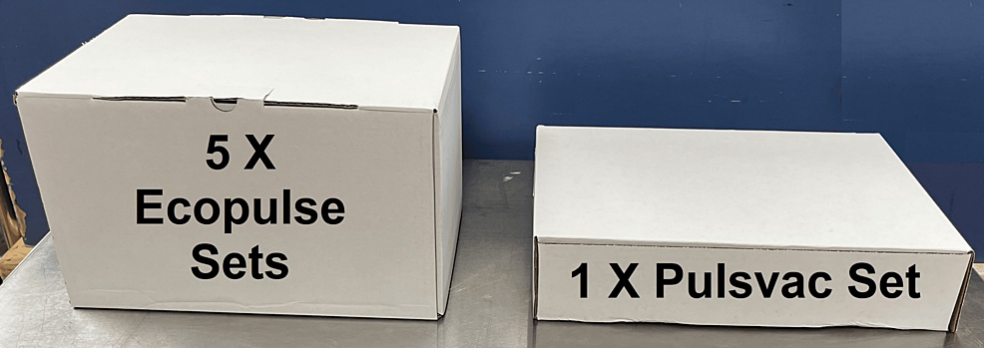
Comparison of packaging of Ecopulse® and Pulsvac® sets Ecopulse® (De Soutter Medical Ltd., Aylesbury, UK); Pulsvac® (Zimmer-Biomet, Warsaw, US).

Another key advantage was the overall physical footprint of the packaging of the power tool operated system was significantly smaller than that of the battery/AC-powered systems. Figure 1 shows a comparison picture of the overall packaging for each product. A box containing a single unit of the AC/battery-powered pulse lavage system is around 1/3 of the size of the power tool operated system that contains five separate pulse lavage units. The manufacturer of the power tool operated system estimates that this will provide a 2.5x increase in storage space. This significant reduction will help create valuable space in operating theatre stores. Additionally, this allows transportation of larger quantities in a single trip. This will then lead to reduced transportation and the overall carbon footprint of the product. The power tool operated system also used clear plastic wrap rather than rigid plastic which has a lower carbon footprint.

The power tool operated system is primarily compatible with the manufacturers' power tools. Different models and adaptors are available to allow compatibility with Stryker®, Linvatec®/Hall®, Aesculap® and Synthes® power tools. Although this encompasses most surgical power tools on the market it may not be possible to use this system in all trusts due to compatibility issues with existing systems. When a power tool operated system is not possible, we strongly advocate the use of AC-powered pulse lavage systems.

## Discussion

Healthcare services account for 4-5% of the total UK carbon emissions [8]. A large proportion of this comes from surgery. Alam et al. [9] estimated that 154 kg of waste was generated per day by the average orthopaedic team, which was 60% greater than the next closest speciality. Additionally, Pegg et al. [10] calculated the average volume of waster per primary hip replacement was 10.9 kg, of which single-use devices represented a significant proportion. Clearly, reducing the wasted disposable items used within each operation represents a key factor in reducing the overall carbon footprint of orthopaedics. This is particularly important in high-volume operations such as total hip replacements. To date, this is the only paper to review the environmental impact of disposable pulse lavage systems in arthroplasty. By introducing a power tool driven pulse lavage system, we have made significant potential carbon savings. However, it is important to project and consider these benefits nationally and globally. The UK National Joint Registry reported 162,818 total knee and hip replacements were performed in 2021 [11], whilst Culliford et al. [6] project that in 2035 over 214,000 hip and knee replacements will be performed in the UK. Using this projection for 2035 and our carbon emissions estimations, we predict that this would generate 925 Tonnes of CO2e if battery operated pulse lavage systems are used. By using power tool operated systems, this figure will be reduced to 361 Tonnes of CO2e, saving 564 Tonnes of CO2e per year. We estimate that this is equivalent to nearly 1.6 million miles driven by the average passenger car (emission factor for average car 0.3472 kgCO2e/mile. In addition, our economical analysis would suggest that each trust could make significant cost savings.

We have only considered disposable pulse lavage systems. Reusable models are available (Palavage®, Hereus Medical) and could represent even greater carbon savings. However, trusts would have to factor in the sterilisation process, which will impact any savings and may not be possible for every orthopaedic unit.

Limitations

We have considered three types of pulse lavage systems commercially available in the UK. Our search has highlighted these broadly represent the pulse lavage systems models available on the market. However, the calculations on cost and carbon emissions will vary between the various products available. This is particularly the case for carbon footprinting, which is dependent on the weights of the individual raw materials used. We were only able to weigh the gross elements of the materials used and were not able to fully break down each unit. Consequently, our carbon footprinting figures can only be considered as gross estimations. Additionally, we have shown that the carbon footprint for transportation from the place of manufacture plays a significant role. The power tool operated system is manufactured and distributed from the UK significantly reduced the overall carbon footprint. Countries outside of the UK will need to take this into consideration when evaluating the pulse lavage options.

Lastly, we have compared the list price for each model; however, the actual price that each trust will be offered will likely vary and is related to several factors such as volume and pre-existing manufacturer deals etc. We advise procurement teams to take the time to discuss with each manufacturer to determine the most appropriate deal for them.

Overall, due to the limitation in our reported outcomes, they should only be used as estimations, and we recommend that a formal analysis of all the products available on the marker be taken locally as results may differ. We hope this article will help guide the process of analysing the carbon footprint of disposable pulse lavage systems and other surgical instruments.

## Conclusions

It is important to consider the environmental implications of medical products used in high-volume operations such as joint replacement surgery. This is especially the case for single-use items such as pulse lavage. This review highlights that power tool operated systems present significant opportunities to reduce the carbon footprint of joint replacement operations. However, for hospitals in which this is not suitable, AC-powered pulse lavage systems are offered as an alternative by many manufacturers and represent a less wasteful option compared to battery-powered counterparts.

The key learning point from this review is that there is a growing market for “green” alternatives in surgical instruments, providing a significant opportunity for surgeons to make carbon emission savings. Taking the time to review the whole carbon footprint of a product can highlight significant opportunities for savings. These calculations are simple and can be done easily by any healthcare professional or procurement team.
